# Implementing and assessing a service to demonstrate public impact of faculty research in news and policy sources

**DOI:** 10.5195/jmla.2019.709

**Published:** 2019-10-01

**Authors:** Caitlin J. Bakker, Jenny McBurney, Katherine V. Chew, Melissa Aho, Del Reed

**Affiliations:** Associate Librarian, University of Minnesota Health Sciences Libraries, University of Minnesota, Minneapolis, MN, cjbakker@umn.edu; Assistant Librarian, University Libraries, University of Minnesota, Minneapolis, MN, jmcburne@umn.edu; Associate Librarian, University of Minnesota Health Sciences Libraries, University of Minnesota, Minneapolis, MN, chewx002@umn.edu; Library Professional, University of Minnesota Health Sciences Libraries, University of Minnesota, Minneapolis, MN, aho@umn.edu; Library Professional, University of Minnesota Health Sciences Libraries, University of Minnesota, Minneapolis, MN, reedx013@umn.edu

## Abstract

**Background:**

As the need to demonstrate research impact increases, faculty are looking for new ways to show funders, departments, and institutions that their work is making a difference. While traditional metrics such as citation counts can tell one part of this story, these metrics are focused on the academic sphere and often miss the wide-ranging public impact that research can have in areas such as the news or policy documents.

**Case Presentation:**

This case report describes how one library piloted and established the Policy & News Media Impact Service, where librarians generate reports for faculty members of the University of Minnesota Academic Health Center that tracks citations of their research in governmental and organizational policies as well as local, national, and international news media. Workflows of, resources used in, and faculty feedback on the service are described.

**Conclusions:**

This Policy & News Media Impact Service pilot was successful and resulted in the establishment of a permanent service that is available to all departments in the Academic Health Center. Faculty feedback indicated that the service was valuable in demonstrating the public impact of their research.

## BACKGROUND

Researchers, universities, and funding agencies show growing interest in demonstrating not only the academic impact of research through publications and citations, but also the public impact of that research. Since 2009, the seven United Kingdom Research Councils have required an “Impact Summary” and “Pathways to Impact” portion for all grant applications, highlighting how the research project will benefit individuals both within and outside of academia and providing a strategy to engage those potential beneficiaries [1]. In the United States, one of the two merit review criteria for all National Science Foundation proposals is broader impacts, which is “[t]he potential to benefit society and contribute to the achievement of specific, desired societal outcomes” [2].

As a result, researchers are increasingly encouraged to engage with mass media and policy makers to promote the widespread impact of their work and to increase the likelihood of future funding opportunities [3, 4]. Greenhalgh et al. note that “researchers are increasingly expected to be accountable and produce value for money, especially when their work is funded from the public purse” [5]. This need to communicate value is particularly notable in the health sciences, where media coverage is strongly correlated with public perceptions of health-related policy decisions [6], demand for health services [7, 8], and attitudes toward illness [9, 10]. Researchers have described the importance of effective media communication as a means of changing health behaviors at both the individual and community levels [11]. Both within disciplines and journals, tracking and discussion of media impact are becoming more commonplace [12, 13].

The need to develop models to reflect the diverse range of potential impact of research, and subsequent mechanisms to measure that impact, has been well documented, as have the challenges associated with this work. As Graham et al. noted: “competing interests among affected stakeholders can result in a lack of consensus on what constitutes value and what should be measured in order to demonstrate impact” [14]. One systematic review of methodological frameworks in health care research outlined five major impact categories: primary research-related impact, including dissemination and knowledge transfer; influence on policy making; health and health systems impact; health-related and societal impact; and broader economic impacts [15]. These five major categories were subdivided into sixteen impact subgroups and included eighty different metrics to reflect these concepts of impact.

Librarians are increasingly offering research impact and evaluation services. A recent survey by Gutzman et al. highlighted the breadth of services offered by North American libraries, ranging from small, informal services offered by a single embedded librarian to robust models involving multiple full-time librarians dedicated to metrics-related work [16]. The results of that survey were supported by a scan of Association of Research Libraries member libraries, which found that 93.9% of member libraries offered at least some support for research impact metrics, most commonly web pages, standalone workshops, workshop series, and individual consultations on research impact metrics [17]. Of those supporting research-impact work, most efforts were supported by individual liaison librarians or scholarly communications librarians.

There are notable examples of robust research-impact frameworks developed from an information professional’s perspective, perhaps most well-established of which is the Becker Medical Library Model for Assessment of Research Impact [18]. Such frameworks advocate for a range of measures to be used in order to best approximate the diverse nature of research impact. However, it is important to note that the majority of public-facing services that academic libraries provide in this area have focused on a smaller subsection of specific tools and measures or on enhancement of discoverability of research outputs [18, 19]. While citation in policy, legislation, and clinical practice guidelines is recognized as an indicator of clinical implementation of research findings, both in libraries and the larger research evaluation community [18, 20], the methods for capturing and demonstrating this impact and related impact are not uniform, and their provision as a formalized service in the context of academic libraries is not widespread [17].

This case report outlines how one library developed the Policy & News Media Impact Service for faculty in its Academic Health Center, including its Medical School, School of Public Health, and College of Pharmacy. Beginning as a small, informal offering, this service was formalized as a yearlong pilot that was later expanded into an established service. The pilot was assessed through a survey of participants.

## CASE PRESENTATION

The University of Minnesota Health Sciences Libraries has an emerging suite of services focusing on bibliometrics that was first introduced in 2014 [21–24]. However, this focus on citation-based metrics in an academic space did not fully reflect the impact of research, particularly for researchers who are focused on influencing clinical practice, policy decisions, or educational practice. In 2015, the Health Sciences Libraries began experimenting with an ad hoc pre-pilot service designed to provide data points on impact outside of traditional research impact metrics, including citations in policy documents and media coverage of research for a limited number of academic departments.

Twelve resources were identified during the pre-pilot phase and later expanded to nineteen resources through the pilot phase (Table 1). These resources included a mixture of subscription-based tools, such as ProQuest News & Newspapers and Factiva, and publicly available resources, such as OpenGrey, covering a mixture of policy and news media materials. The team decided early on that social media platforms such as Facebook and Twitter would not be included due to issues surrounding scalability.

**Table 1:** Subscription and publicly available resources

	Policies and grey literature	News and media	Social media
Subscription-based resources			
Access UN	X		
Factiva		X	
Foundation Directory Online	X		
Gale Student Resources in Context*		X	
LexisNexis Academic	X	X	
PolicyFile	X		
ProQuest Congressional Publications & Executive Branch Documents	X	X	X
ProQuest News & Newspapers*		X	
Publicly available resources			
Altmetric Explorer*†	X	X	X
Australian Policy Online	X		
DocuTicker‡	X		
Duck Duck Go*	X	X	X
Global Voices		X	
Google*§	X	X	X
Grey Literature Report**	X		
Index to Current Urban Documents	X		
OpenGrey	X		
Popline	X		
World News Network		X	

* Core resource prioritized due to its coverage and utility.

† Users must request a personal account from Altmetric. These accounts are granted at the discretion of that company.

‡ As of February 2016, this tool is no longer being updated.

§ Searches were restricted using domain and format limits.

** As of January 2017, this tool is no longer being updated.

Through creating three pre-pilot reports, we developed a template to most effectively organize and communicate the results of the search (supplemental Appendix A). The template is organized at a broad level—policies, guidelines, and government documents, and media coverage—and then subdivided into different categories, each of which includes a list of relevant citations. This is prefaced with an executive summary that provides an overview of the results and highlights notable references in policy or news media.

**Figure 1:**
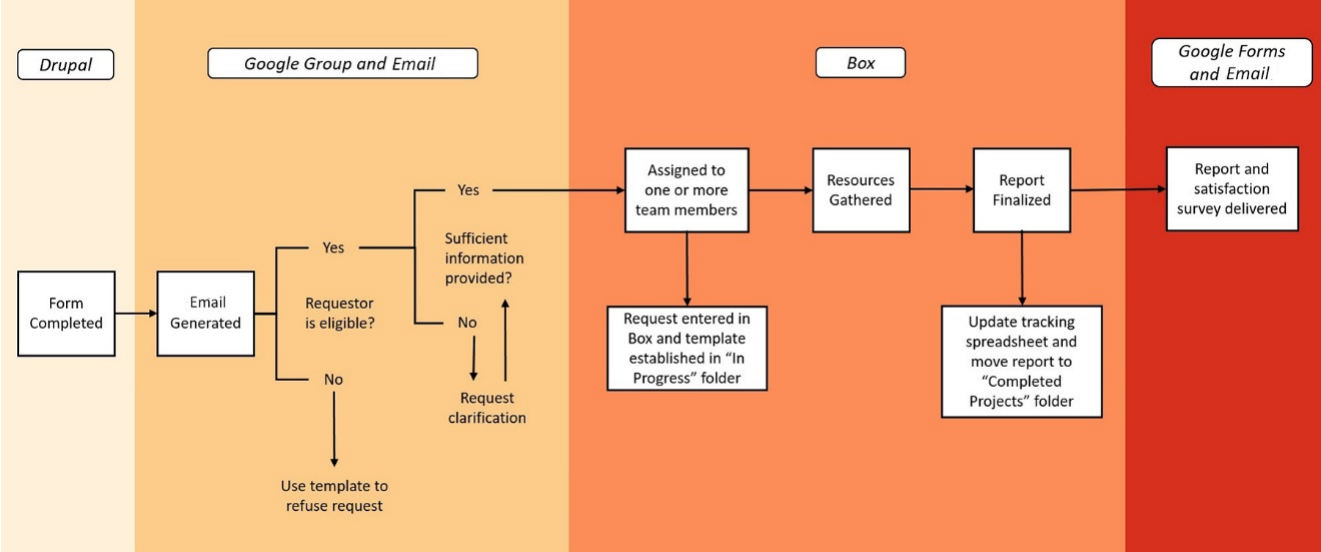
Workflow to ingest requests and produce reports

Following the success of this ad hoc pre-pilot service, we developed a charter for a pilot service, which ran from December 2016 to November 2017 and was officially launched to faculty in February 2017. The pilot charged a team of four librarians, including three from the health sciences and one from the social sciences, to formalize the processes and policies associated with this service, including the refinement of associated activities, tools, and workflows (Figure 1).

We developed an online intake form in which faculty could indicate the scope and intended use of the report as well as provide links to or lists of their relevant grants or publications. Faculty could choose to provide their entire curriculum vita or focus on a subset of publications (e.g., those from the last five years, those focused on a particular research interest). We placed no limits on the number of publications that could be included, although other research outputs, such as conference presentations, were not included.

The intake form triggers an email to the team and notifies the requestor of receipt. Once the service request is received, it is triaged to ensure sufficient information is provided and that the requestor is eligible for the service, after which a project template is established under the requestor’s name in our Box instance. Box is a secure, cloud-based storage system, subscribed to via the university, where data are encrypted and two-factor authentication is required to access all reports. Access is limited to collaborators, and all previous versions of reports are maintained in the system. The university’s instance of Box was selected for this service due to its integration with the Microsoft Office suite of tools as well as its ability to facilitate collaboration and version control. Once produced, the Word document housed in Box is transmitted to the requestor as a portable document format (PDF) file, and the completed report is moved from the “in process” to “completed” folder in our Box instance.

During the pilot phase throughout 2017, eligibility was limited to faculty in five departments: Family Medicine & Community Health (Medical School), Pediatrics (Medical School), Epidemiology & Community Health (School of Public Health), Pharmaceutical Care & Health Systems (College of Pharmacy), and the Center for Bioethics. These departments were selected due to their potential policy impact and their focus on clinical research and community engagement. To make a request, at least one individual was required to be a faculty member in one of these departments. Reports were completed within two weeks of a request, except where other arrangements were made directly with the requestor, and we were committed to devoting no more than twenty hours to the completion of any single report.

Our work was supported through biweekly meetings that served to discuss particular strategies or points of challenge for individual requests, as well as to facilitate broader communication regarding the service. Search tips for the various resources were recorded in a shared document, in which emerging tools of interest were also recorded. We chose to specialize in particular tools and areas rather than attempt to build capacity across a range of resources. As a result, newer team members often split responsibilities in completing reports rather than complete reports independently.

Throughout the pilot, we completed fourteen reports for twelve faculty. Faculty were most often associated with the Departments of Family Medicine & Community Health and Epidemiology & Community Health. The reports were predominantly requested for the purposes of supplementing promotion and tenure dossiers or for grant applications. Requestors were chiefly interested in information regarding policy coverage, followed by news and media coverage, and finally by blog posts and other web content. Upon receiving their reports, all recipients were asked to complete a survey of their satisfaction and to offer suggestions for further improvement of the service (supplemental Appendix B). Eleven of the twelve recipients completed the survey.

Ten of the 11 respondents indicated that they would recommend the service to a colleague, while the remaining respondent indicated that they may recommend the service. Most (82%) respondents found the report to be very useful for their intended purposes, with the remaining (18%) respondents indicating that they found the report to be somewhat useful for their purposes. When asked to rate the ease of engaging with various aspects of the service, most respondents indicated that they found it very easy to find the service web page or online form, to complete the online form, to work with the team members, and to understand the final report (Table 2).

**Table 2:** Perceived ease of engaging with each aspect of the service

	Very difficult	Somewhat difficult		Somewhat easy		Very easy		N/A	

	n	n	%	n	%	n	%	n	%
Find the web page?	0	0	—	1	9%	8	73%	2	18%
Complete the form?	0	0	—	0	—	10	91%	1	9%
Work with the group?	0	0	—	0	—	11	100%	0	—
Understand the report?	0	1	9%	1	9%	9	82%	0	—

Respondents were also asked to provide free-text answers on their opinions of the service and areas for improvement. The responses were overwhelmingly positive. One respondent referred to their report as “mak[ing] a really strong contribution to showing the impact of my work to a potential funder,” while another described it as “an incredible quantification of my work that will positively impact my ability to go up for promotion.” One respondent summarized the service as “pure gold for helping to understand, and share, the impact of my work.”

Although faculty feedback on areas for improvement was limited, there were some suggestions to clarify and improve the reports. There was interest in providing greater information on which publications had been most influential and providing more explicit methodology. We are investigating whether incorporating changes to meet these needs would be feasible within the resource constraints in place.

Following the success of the pilot, the service was established as a Health Sciences Libraries’ offering. Its eligibility was expanded to the entire Academic Health Center, which includes the Medical School, School of Nursing, School of Public Health, College of Veterinary Medicine, College of Pharmacy, School of Dentistry, and associated research centers. We continue to review and refine the policies and procedures established during the pilot on an ongoing basis.

## DISCUSSION

Health care administrators and policy makers are increasingly concerned with assessing health sciences research impact on the economy, society, media, and the public at local, regional, national, and international levels [25, 26]. Given the current funding environment and international trends, this need will likely become increasingly important. For those faculty whose work may have practice and policy implications, and whose influence is not fully captured through bibliometrics [27–29], this growing understanding of research impact creates opportunities to more fully describe the outcomes of their research.

The goal of the Policy & News Media Impact Service is to describe a broader scope of the research impact of individual scholars in public policy and media. This service provides faculty with a means of demonstrating impact beyond citation counts for a variety of purposes, including promotion and tenure dossiers and grant applications. Such a service expands a library’s traditional offerings in the research metrics space to reflect a broader understanding of the meaning of research impact. Utilizing existing library resources reduces potential costs, while allowing those resources to provide greater value, as they may be underutilized for the purpose of research impact assessment. Libraries continue to consider ways to further articulate their value, both to the campus and broader communities. Highlighting the multipurpose nature of our collections—and the expertise of our librarians in using those resources in novel ways—is one way libraries can tell that story.

The team producing these reports has not exceeded the allotted time for completing any of the reports. However, the reports nevertheless represent a significant investment of staff resources, not only in completing the reports, but also in developing the necessary skills. Because the utilized resources are not in the health sciences, medical librarian search skills may not be transferable to this work. We found that by having different team members focusing on specific tools or areas, such as government publications or newspapers, we were able to streamline workflows by minimizing the number of resources with which any one individual would need to develop fluency, while at the same time ensuring efforts were not duplicated. While reports have been completed for individuals and small groups, as of yet, no research center or department has requested this service. If we received such a request, timelines and workflows would be adjusted to account for the increased workload that would be expected from a more broadly scoped request.

When considering how works are represented in the template, we grappled with the nature of how we would count these nontraditional items. As outlined by Yu, there are two primary ways to count altmetrics activities: number of unique users (NUU) and number of posts (NP) [30]. When one user makes multiple posts on a topic, the NP would be larger than the NUU. While our service does not include social media, the issue of what constitutes a reference surfaced with regard to syndicated news items, where one item authored by one individual is subsequently reproduced in a variety of other publications. We chose to count the original item rather than its reproductions. The decision of how to count items may appear trivial, but as Yu notes, the NP altmetrics indicators—including news, blogs, and policy—have a “moderate to low correlation with NUU, indicating that they convey different message[s]” [30]. While the example here is not an exact comparator to Yu’s study, it highlights the level of detail that must be considered when providing these services and the potential impact of these decisions.

We chose not to curate reports, meaning we include all aspects of broader engagement, including when a researcher’s work is criticized. As a team, we philosophically believe impact and agreement are not synonymous. This stance is in line with previous discourse on negative citation in bibliometrics, namely that “it is somewhat of an achievement to have one’s work noticed by others, even if negatively; work deemed substandard or negligible is seldom cited at all” [31]. We believe impact is seen through substantive engagement with the original content. This belief contributed to the decision to exclude social media from these reports. Beyond concerns regarding scalability, there were concerns regarding the extent to which social media reflects impact on society. Due to the succinct nature of social media posts, these activities often act as “pointers to research” rather than providing substantive commentary or reflection [32]. While we believe that there is value in social media analysis of public attention and interest, the scope of social media activities did not align with the intended purpose of our service.

There was interest in determining whether a single tool or more automated approach to report production would be possible. While numerous tools in the altmetrics space appear to operate in a similar fashion, our analysis of those tools led us to conclude that none provides the comprehensive, customized end product that is of most value to our users [33]. Due to the ephemeral nature of the materials being sought, no tool can claim to be comprehensive. Several tools do provide user-friendly interfaces through which to access the available content, which may benefit individual institutions. The costs of subscription access must be balanced against organizational needs and constraints, particularly in the context of necessary staff time.

Marketing the service emerged as a primary challenge. Despite initial concerns regarding scalability following the broad advertisement of the service, when asked how faculty became informed of the service, only one responded that they had seen the libraries’ marketing efforts, whereas most were informed of the service by colleagues. The assumption that personal experience or recommendation is the most compelling marketing strategy in this scenario is reinforced by multiple requests for reports, where we update existing reports to show newer references in policy and media, or to include references to publications produced after the last report. These most commonly occur as faculty are preparing their promotion and tenure dossiers, and want to ensure that the dossiers are as comprehensive and current a representation of impact as possible.

We recognize that this service captures one aspect of research impact and is not representative of the full scope of the influence of a researcher’s work. It should also be acknowledged that policy impact is often a medium- to long-term measure [29]. Our approach maintains a fundamental flaw in research impact assessment, described by Stern as the “linking of a particular publication to a particular activity or policy decision…[I]mpact should be interpreted much more subtly and broadly to link bodies of work and disciplinary or collaborative activity to outcomes understood from a more nuanced and deeper perspective” [34]. Our approach does not necessarily reflect the full scope of a researcher’s influence. Moreover, as faculty hold multifaceted positions, these reports do not reflect their contributions in teaching, service, or clinical work or the administrative responsibilities that these individuals may have.

Like all measures of research impact, the reports must be contextualized within the full scope of an individual’s work and within their discipline and career stage. Despite these caveats, we believe that this service offers faculty additional data points and that these reports can function as tools for self-advocacy as faculty demonstrate the significance of their accomplishments and potential to administrators, funding agencies, and the broader community.

## SUPPLEMENTAL FILES

Appendix A Report template

Appendix B Evaluation form

## 

**Caitlin J. Bakker, AHIP**, cjbakker@umn.edu, http://orcid.org/0000-0003-4154-8382, Associate Librarian, University of Minnesota Health Sciences Libraries, University of Minnesota, Minneapolis, MN

**Jenny McBurney**, jmcburne@umn.edu, http://orcid.org/0000-0003-4081-6066, Assistant Librarian, University Libraries, University of Minnesota, Minneapolis, MN

**Katherine V. Chew**, chewx002@umn.edu, http://orcid.org/0000-0003-0788-3179, Associate Librarian, University of Minnesota Health Sciences Libraries, University of Minnesota, Minneapolis, MN

**Melissa Aho**, aho@umn.edu, Library Professional, University of Minnesota Health Sciences Libraries, University of Minnesota, Minneapolis, MN

**Del Reed**, reedx013@umn.edu, Library Professional, University of Minnesota Health Sciences Libraries, University of Minnesota, Minneapolis, MN

## References

[1] UK Research and Innovation Pathways to impact [Internet].

[2] National Science Foundation (2018). Chapter III - NSF proposal processing and review. Proposal & award policies & procedures guide [Internet].

[3] Jaques H (2011). Get your research reported well in the news. BMJ.

[4] Brownell KD, Roberto CA (2015). Strategic science with policy impact. Lancet.

[5] Greenhalgh T, Raftery J, Hanney S, Glover M (2016). Research impact: a narrative review. BMC Med.

[6] Fredriksson M, Tiainen A, Hanning M (2015). Regional media coverage influences the public’s negative attitudes to policy implementation success in Sweden. Heal Expect.

[7] Gafson AR, Giovannoni G (2014). CCSVI-A. a call to clinicians and scientists to vocalise in an Internet age. Mult Scler Relat Disord.

[8] Almomani B, Hawwa AF, Goodfellow NA, Millership JS, McElnay JC (2015). Pharmacogenetics and the print media: what is the public told?. BMC Med Genet.

[9] Previte J, Gurrieri L (2015). Who is the biggest loser? fat news coverage is a barrier to healthy lifestyle promotion. Health Mark Q.

[10] MacLean A, Sweeting H, Walker L, Patterson C, Räisänen U, Hunt K (2015). “It’s not healthy and it’s decidedly not masculine”: a media analysis of UK newspaper representations of eating disorders in males. BMJ Open”.

[11] Thrasher JF, Kim SH, Rose I, Craft MK (2015). Media coverage of smoke-free policies after their innovation. J Health Commun.

[12] Dunn J, Koo J (2014). Social media impact factor: the top ten dermatology journals on Facebook and Twitter. Dermatol Online J.

[13] Roberts J (2014). Measuring the social media impact of your headache article. Headache.

[14] Graham K, Chorzempa H, Valentine P, Magnan P (2012). Evaluating health research impact: development and implementation of the Alberta Innovates - Health Solutions impact framework. Res Eval.

[15] Rivera S, Kyte DG, Aiyegbusi OL, Calvert MJ (2017). Assessing the impact of healthcare research: a systematic review of methodological frameworks. PLOS Med.

[16] Gutzman KE, Bales ME, Belter CW, Chambers T, Chan L, Holmes KL, Lu YL, Palmer LA, Reznik-Zellen RC, Sarli CC, Suiter AM, Wheeler TR (2018). Research evaluation support services in biomedical libraries. J Med Libr Assoc.

[17] Bakker C, McBurney J Big splashes & ripple effects: research impact services across university libraries [Internet]. https://conservancy.umn.edu/handle/11299/188632.

[18] Sarli CC, Dubinsky EK, Holmes KL (2010). Beyond citation analysis: a model for assessment of research impact. J Med Libr Assoc.

[19] Lewis R, Sarli CC, Suiter AM (2015). SPEC kit 346: scholarly output assessment activities [Internet].

[20] Grant J (2015). The nature, scale and beneficiaries of research impact: an initial analysis of Research Excellence Framework (REF) 2014 impact case studies [Internet].

[21] Braun S (2015). Manifold: a custom analytics platform to visualize research impact. Code4Lib [Internet].

[22] Bakker C Contextualizing impact: the University of Minnesota’s Manifold Initiative.

[23] Bakker C, Chew K Evaluative bibliometrics meets the CTSI [Internet]. http://hdl.handle.net/11299/180871.

[24] Chew K, Bakker C Adventures in bibliometrics: research impact and the CTSI [Internet]. http://hdl.handle.net/11299/194375.

[25] Sa CM, Kretz A, Sigurdson K (2013). Accountability, performance assessment, and evaluation: policy pressures and responses from research councils. Res Eval.

[26] Terama E, Smallman M, Lock S, Johnson C, Austwick MZ (2016). Beyond academia – interrogating research impact in the research excellence framework. PLOS ONE.

[27] Chubb J, Reed M (2018). The politics of research impact: academic perceptions of the implications for research funding, motivation and quality. Br Politics.

[28] Derrick GE, Haynes A, Chapman S, Hall WD (2011). The association between four citation metrics and peer rankings of research influence of Australian researchers in six public health fields. PLOS ONE.

[29] Brownson RC, Eyler AA, Harris JK, Moore JB, Tabak RG (2018). Getting the word out: new approaches for disseminating public health science. J Public Health Manag Pract.

[30] Yu H (2017). Context of altmetrics data matters: an investigation of count type and user category. Scientometrics.

[31] White HD (2004). Citation analysis and discourse analysis revisited. Appl Linguist.

[32] Timilsina M, Khawaja W, Davis B, Taylor M, Hayes C (2017). Social impact assessment of scientist from mainstream news and weblogs. Soc Netw Anal Min.

[33] Bakker C, Chew K, McBurney J, Reed D, Aho M Measuring impact with altmetrics: is there one tool to rule them all? [Internet]. http://hdl.handle.net/11299/200727.

[34] Stern N (2016). Building on success and learning from experience: an independent review of the Research Excellence Framework [Internet].

