# Supplementary material for: Implementing and assessing a service to demonstrate public impact of faculty research in news and policy sources
Source: J Med Libr Assoc. 2019 Oct 1;107(4):579–87. doi: 10.5195/jmla.2019.709 (PMC6774553; doi:10.5195/jmla.2019.709)
Supplement: Appendix A [file jmla-107-579-s001.pdf]

JMLA Journal of the Medical Library Association

SC

Supplemental content to
J Med Libr Assoc. Oct;107(4):dx.doi.org/10.5195/jmla.2019.709
www.jmla.mlanet.org
© Bakker, McBurney, Chew, Aho, Reed 2019

Implementing and assessing a service to demonstrate public impact of faculty research in news and policy sources

Caitlin J. Bakker, AHIP; Jenny McBurney; Katherine V. Chew; Melissa Aho; Del Reed

APPENDIX A

Report template

Demonstrating the policy and news media impact of the research of Dr. [full name]

Prepared by the Health Sciences Libraries Policy & News Media Impact Service*
[month] [year]

The research of Dr. [full name] has been referenced [n] times in [n] different policy and news media resources. Dr. [last name]'s work has been referenced in government documents by groups such as [include notable examples here].

Dr. [last name]'s research has been referenced [n] times in [n] popular media channels, including newspapers and websites. Their work has received attention through news outlets such as [insert examples here]. It is likely that this does not represent the full impact of this work as this report is limited to resources that are currently available.

The following report includes lists of citations for policy and news media resources that have cited Dr. [last name]'s work. An index is included, beginning on page [x].

Outline

Policies, guidelines, and government documents – p.
United States (federal) – p.
United States (state and local) – p.
International (federal) – p.
International (state and local) – p.
Organizations, associations, and societies – p.

Media coverage – p.
News and magazines – p.
Websites and blogs – p.
Television, film, and radio – p.
Syndicated news items – p.
Index – p.

* The Policy & News Media Impact Service includes Caitlin J. Bakker, AHIP; Katherine V. Chew; Jenny McBurney; Melissa Aho; and Del Reed. Any questions or comments about the service can be directed to lib-impact@umn.edu.

JMLA Journal of the Medical Library Association

SC
Supplemental content to
J Med Libr Assoc. Oct;107(4):dx.doi.org/10.5195/jmla.2019.709
www.jmla.mlanet.org
© Bakker, McBurney, Chew, Aho, Reed 2019

Policies, guidelines, and government documents

United States (federal)

Group

Citation

United States (state and local)

Group

Citation

International (federal)

Group

Citation

International (state and local)

Group

Citation

Organizations, associations, and societies

Group

Citation

Media coverage

News and magazines

Title

Citation

Websites and blogs

Title

Citation

Television, film, and radio

Title

Citation

Syndicated news items

Citation

JMLA Journal of the Medical Library Association

SC
Supplemental content to
J Med Libr Assoc. Oct;107(4):dx.doi.org/10.5195/jmla.2019.709
www.jmla.mlanet.org
© Bakker, McBurney, Chew, Aho, Reed 2019

Index

Outline....1
Policies, guidelines, and government documents....2
United States (federal)....2
Group....2
United States (state and local)....2
Group....2
International (federal)....2
Group....2
International (state and local)....2
Group....2
Organizations, associations, and societies....2
Group....2
Media coverage....2
News and magazines....2
Title....2
Websites and blogs....2
Title....2
Television, film, and radio....2
Title....2
Syndicated news items....2
