# Supplementary material for: Implementing and assessing a service to demonstrate public impact of faculty research in news and policy sources
Source: J Med Libr Assoc. 2019 Oct 1;107(4):579–87. doi: 10.5195/jmla.2019.709 (PMC6774553; doi:10.5195/jmla.2019.709)
Supplement: Appendix B [file jmla-107-579-s002.pdf]

JMLA Journal of the Medical Library Association

SC

Supplemental content to
J Med Libr Assoc. Oct;107(4):dx.doi.org/10.5195/jmla.2019.709
www.jmla.mlanet.org
© Bakker, McBurney, Chew, Aho, Reed 2019

Implementing and assessing a service to demonstrate public impact of faculty research in news and policy sources

Caitlin J. Bakker, AHIP; Jenny McBurney; Katherine V. Chew; Melissa Aho; Del Reed

APPENDIX B

Evaluation form

1. Was the report useful for your intended purposes?
- o Very useful
- o Somewhat useful
- o Not very useful
- o Not at all useful

2. How easy or difficult were the following?

	Very difficult	Somewhat difficult	Somewhat easy	Very easy	Not applicable
Finding the web page or online form	○	○	○	○	○
Completing the online form	○	○	○	○	○
Working with the group member(s)	○	○	○	○	○
Understanding the final report	○	○	○	○	○

3. How did you hear about this service (please select all that apply)?
- o Email advertisement
- o Library website
- o Recommendation from a colleague or friend
- o Google search result
- o Recommendation from a librarian or library staff person
- o Other: ______________________________

4. Would you recommend this service to a colleague?
- o Yes
- o No
- o Maybe

5. Do you have any other comments or suggestions?

6. If you would like a follow-up response, please enter your email address below.
